# The Significance of Selected Myokines in Predicting the Length of Rehabilitation of Patients after COVID-19 Infection

**DOI:** 10.3390/biomedicines12040836

**Published:** 2024-04-10

**Authors:** Alicja Mińko, Agnieszka Turoń-Skrzypińska, Aleksandra Rył, Katarzyna Mańkowska, Aneta Cymbaluk-Płoska, Iwona Rotter

**Affiliations:** 1 Department of Medical Rehabilitation and Clinical Physiotherapy, Pomeranian Medical University, 71-210 Szczecin, Poland; agi.skrzypinska@gmail.com (A.T.-S.); aleksandra.ryl@pum.edu.pl (A.R.); iwrot@wp.pl (I.R.); 2 Department of Microbiology, Immunology and Laboratory Medicine, Pomeranian Medical University, 70-111 Szczecin, Poland; katarzyna.mankowska@pum.edu.pl; 3 Department of Reconstructive Surgery and Gynecological Oncology, Pomeranian Medical University in Szczecin, 70-111 Szczecin, Poland; aneta.cymbaluk.ploska@pum.edu.pl

**Keywords:** COVID-19, myokine, rehabilitation, SARS-CoV-2

## Abstract

In the context of the global COVID-19 pandemic, understanding the intricate mechanisms of the body’s response to infection and inflammation has become a priority for the medical and research communities. It has been proven that during COVID-19 infection, molecules are secreted—namely organokines, which may directly or indirectly play a role in the pathophysiology of COVID-19. The objective of this study was to scrutinize the potential correlation between the levels of selected myokines (myostatin, agrin, irisin, and myonectin) and the duration of rehabilitation in post-COVID-19 patients. Additionally, the study aimed to investigate whether there is a correlation between the levels of these myokines and the length of hospitalization during COVID-19 treatment. The study was conducted at the Rehabilitation Hospital in Szczecin (Poland). Patients in the study participated in a comprehensive rehabilitation program following COVID-19 treatment. In order to assess the effectiveness of rehabilitation, the following tests were performed: a 6 min walk test with an assessment of exercise tolerance (Borg scale), an assessment of dyspnea severity (mMRC scale), a spirometric assessment of respiratory function, a measurement of arm strength, and an assessment of fatigue using the Fatigue Assessment Scale (FAS). Myokine levels were measured using commercially available enzyme-linked immunosorbent assays (ELISA) according to the manufacturer’s instructions. Statistical analysis was performed using Statistica 13.1 software. Lower concentrations of irisin and myonectin and higher concentrations of myostatin correlated with longer rehabilitation time. Baseline levels of specific myokines in post-COVID-19 patients could play a crucial role in anticipating the duration of rehabilitation. The duration of hospitalization for the infection may influence myokine levels in patients recovering from COVID-19.

## 1. Introduction

In the context of the global COVID-19 pandemic, understanding the intricate mechanisms of the body’s response to infection and inflammation has become a priority for the medical and research communities. While the primary target of SARS-CoV-2, the virus responsible for COVID-19 is the respiratory system, emerging evidence suggests that its impact extends to metabolic and muscular alterations in the body [1,2,3].

COVID-19 infection has been demonstrated to prompt the release of organokines (adipokines, osteokines, myokines, hepatokines, and cardiokines) from adipose tissue, bone, skeletal muscle, liver, and heart, respectively. These molecules may play a direct or indirect role in the pathophysiology of COVID-19 [1,4]. Organokines can exhibit either beneficial or detrimental effects during infection, contributing to inflammation, immune dysregulation, and the onset of sarcopenia [5,6,7]. Moreover, they can serve as biomarkers for monitoring symptoms, gauging disease severity, and predicting outcomes [1].

Myokines, including cytokines and peptides, exert autocrine, paracrine, or endocrine effects and are produced by muscle fibers [8,9,10]. They primarily regulate processes associated with physical activity and mediate anti-inflammatory processes related to exercise [11,12]. Analyzing the myokine profile of patients can offer valuable insights into the trajectory of recovery, potential complications, and optimal rehabilitation strategies. Key myokines such as myostatin, agrin, irisin, and myonectin may play pivotal roles in the pathogenesis, response to infection, and outcomes of COVID-19 patients [1,13,14].

Irisin, induced by physical activity, promotes lipid metabolism and improved metabolism, which is crucial for patients returning to activity after prolonged illness [15,16,17]. Research indicates that irisin may significantly impact the modulation of COVID-19-related genes, resulting in a decrease in genes associated with increased viral infection and an increase in genes that block angiotensin-converting enzyme 2 receptor activity [17,18]. Myonectin, also known as CTRP15, regulates glucose and lipid metabolism, holding importance for patients with metabolic disorders induced by COVID-19 or bed rest. It plays a crucial role in regulating fatty acid metabolism, lowering blood levels of free fatty acids via increased uptake into adipose tissue and the liver. Myonectin concentration rises during physical activity [19,20,21]. Agrin is essential for neuromuscular junction formation, potentially impacting muscle function and coordination during recovery [22]. Myostatin, a negative regulator of skeletal muscle growth, inhibits protein synthesis, leading to skeletal muscle atrophy. Monitoring its levels is vital for assessing muscle mass loss and determining physiotherapy needs [23,24,25].

The objective of this study was to scrutinize the potential correlation between the levels of selected myokines and the duration of rehabilitation in post-COVID-19 patients. Additionally, the study aimed to investigate whether there is a correlation between the levels of these myokines and the length of hospitalization during COVID-19 treatment.

## 2. Materials and Methods

### 2.1. Patients

This study was conducted at the St. Karol Boromeusz Rehabilitation Hospital in Szczecin, Poland, from May 2021 to September 2022. The study included 171 participants staying at the post-COVID-19 rehabilitation unit, where in-patient rehabilitation of patients after SARS-CoV-2 infection is performed in accordance with the guidelines of the National Health Fund in Polnad (NFZ), as specified in Order No. 42/2021/DSOZ of the President of the NFZ dated 5 March 2021 [26].

The qualification for the post-COVID-19 in-patient rehabilitation program was based on the guidelines of the National Health Fund in Poland [26]. Qualification for rehabilitation was performed by a physician specializing in medical rehabilitation. Patients with post-COVID-19 complications were qualified for rehabilitation, which was assessed using the Post-COVID-19 Functional Status (PCFS) scale (score 1–4), the Medical Research Council (score < 5), and the modified Medical Research Council (score ≥ 1). The Post-COVID-19 Functional Status Scale is a five-point scale used to identify patients with functional limitations in many aspects of health after COVID-19 [27]. The Medical Research Council is a scale used to test muscle strength. The score ranges from 0 to 5, where 0 is no muscle tone, and 5 is normal muscle strength [28]. The modified Medical Research Council is a five-point scale used to assess the severity of dyspnea. A score of 0 indicates shortness of breath only during strenuous exercise, whereas a score of 4 indicates shortness of breath that prevents leaving the house [29].

Other inclusion criteria were age > 18 years, confirmed diagnosis of COVID-19 with a positive polymerase chain reaction test for SARS-CoV-2, a period of no more than 12 months after completion of COVID-19 treatment, and consent to the collection of biological material (blood). The end of COVID-19 treatment was defined as the date of completion of home isolation, hospital discharge, or isolation. The diagnostic test required to qualify for rehabilitation was also a chest X-ray with a description taken after the end of treatment in the acute phase of the disease.

**Figure 1:**
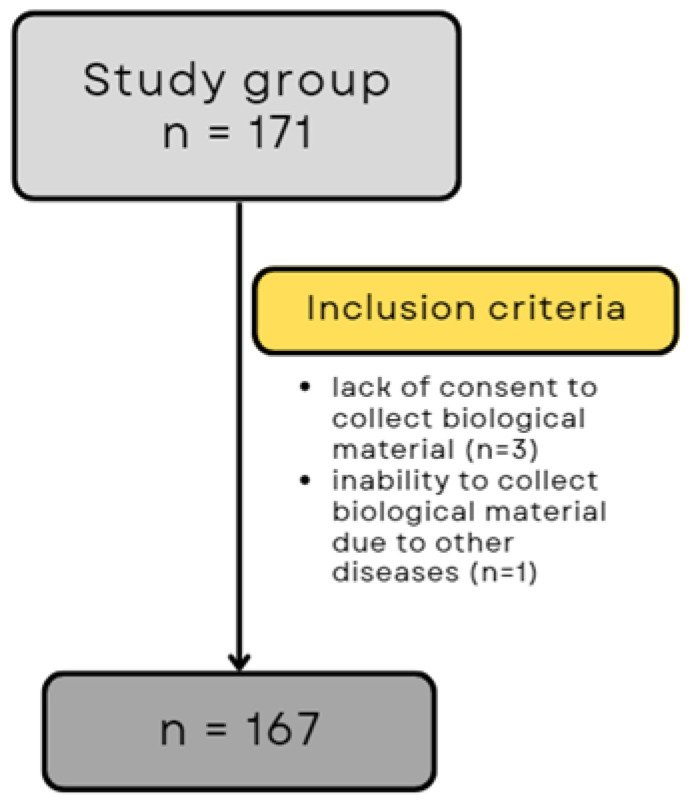
Flowchart of patient qualification for the study.

Exclusion criteria were age <18 years, refusal to participate in the study, lack of consent for the collection of biological material (blood), and diseases that prevented consent to the study or understanding the nature of the study and the conditions for participation. Finally, taking into account all inclusion and exclusion criteria, 167 patients participated in the study (Figure 1).

Each patient gave written informed consent to participate in this study and to use data from their medical records. Every effort has been made to protect the privacy and anonymity of patients. The study was conducted in accordance with the current version of the Declaration of Helsinki. Approval to conduct the study was obtained from the Bioethics Committee of the Pomeranian Medical University in Szczecin (decision no. KB-0012/15/2021).

### 2.2. Study Process

Patients in the study participated in a comprehensive rehabilitation program following COVID-19 treatment. The comprehensive rehabilitation program included breathing exercises, aerobic training, and strength and endurance training. The rehabilitation classes were held six times a week, Monday through Saturday. 

Each day, patients performed the following:

- Breathing exercises (active breathing exercises, active breathing exercises with resistance, learning effective coughing, and clearing the airways, time: 30 min);
- Aerobic exercise (stair climbing, outdoor walking, continuous/interval exercise on a bicycle ergometer, assessment of exercise tolerance by monitoring oxygen saturation (pulse oximeter), and perceived exertion on the Borg scale; gradual increase in intensity by 5–10%, time: 90 min);
- Strength and endurance training (individualized training based on 1 repetition maximum (1 RM) and exercise tolerance (assessment of desaturation); load: 70–85% of 1RM; volume: 3 sets of 8–12 repetitions; rest: 1–2 min; progression: 60–70% of 1RM, time: 30 min).

Throughout the rehabilitation period, the patient was under medical, nursing, and physiotherapy care. The minimum rehabilitation period was 2 weeks. The decision to prolong rehabilitation (up to a maximum of 6 weeks) was made by the treating physician based on a comparison of the current test results with those obtained before the start of rehabilitation. This included an exercise test (6 min walk test) with an assessment of exercise tolerance (Borg scale), an assessment of dyspnea severity (mMRC scale), a spirometric assessment of respiratory function, a measurement of arm strength, and an assessment of fatigue using the Fatigue Assessment Scale (FAS).

Spirometry was performed using a BTL-08 Spiro Pro spirometer (BTL Industries, Newcastle-under-Lyme, UK). The following parameters were determined: forced expiratory volume in one second (FEV1), forced vital capacity (FVC), and the FEV1/FVC ratio. Results were expressed as a percentage of the patient’s predicted normal values, which were automatically calculated based on age, sex, height, weight, and ethnicity. The FEV1/FVC ratio is presented as an absolute value. The ECCS/ERS 1993 reference values were used for interpretation of the spirometry data. All spirometric measurements were performed according to the standard recommendations of the American Thoracic Society (ATS) and the European Respiratory Society (ERS) [30].

The six-minute walking test (6MWT) was performed according to the American Thoracic and European Respiratory Society standards [31]. The 6MWT was measured along a straight, paved corridor 30 m in length. The distance covered by the patient in 6 min was measured. The results were expressed as an absolute value in meters. At the end of the 6MWT, fatigue was measured using the Borg scale (from 6 to 20). The number 6 on the Borg scale indicates no fatigue, and 20 indicates maximum fatigue [32].

A Charder MG 4800 hand dynamometer (Charder Electronic Co., Taichung, Taiwan) was used to measure arm strength. During the task, participants sat comfortably in a chair with arms adducted and elbow flexed at 90°. Measurements were taken for the dominant hand. The measurement data were read from a digital display with an accuracy of 0.1 kg. The maximum compressive force (kg) was considered.

The mMRC scale, developed by the British Medical Research Council, is used to assess dyspnea [29]. The scale consists of 5 statements with scores ranging from 0 to 4. A score of 0 is given if dyspnea occurs only during heavy physical exertion. A score of 4 is given to patients whose dyspnea prevents them from leaving the house or occurs when dressing or undressing. 

The FAS is a scale that assesses fatigue [33]. It consists of 10 questions with answers ranging from 1 (never) to 5 (always). The total FAS score ranges from 10 to 50 and increases with fatigue. A FAS score > 22 indicates significant fatigue, while a score ≥ 35 indicates extreme fatigue. 

An interview was also conducted with each subject on the day of rehabilitation to obtain sociodemographic data. Information on disease course, treatment, and comorbidities was obtained from medical records. Peripheral venous blood samples were taken on the day of rehabilitation.

In this paper, the term hospitalization refers to the length of time a patient was hospitalized during SARS-CoV-2 infection. The term rehabilitation refers to the length of time a patient stays in a rehabilitation hospital after a COVID-19 infection.

### 2.3. Blood Sampling

Peripheral venous blood samples were obtained from each patient. Blood samples were collected in tubes containing ethylenediaminetetraacetic acid (EDTA). Plasma was prepared from the blood by centrifugation, frozen in aliquots, and stored at −80 °C until laboratory analysis.

### 2.4. ELISA Tests

Samples were thawed at room temperature prior to testing. Laboratory analyses for irisin (Human irisin ELISA Kit, Sun Red Biotechnology Company, Shanghai, China), myonectin (Human C1q and tumor necrosis factor-related protein 15 ELISA Kit, Sun Red Biotechnology Company, Shanghai, China), agrin (Human agrin ELISA Kit, Sun Red Biotechnology Company, Shanghai, China), and myostatin (Human myostatin ELISA Kit, Sun Red Biotechnology Company, Shanghai, China) were performed using commercially available enzyme-linked immunosorbent assays (ELISAs) according to the manufacturer’s instructions. Plates containing standards were first prepared according to the manufacturer’s instructions. Biotin-labeled antibodies, test material, and streptavidin were then added. The volume of test material and reagents depended on the parameter to be determined. The prepared plates were incubated at 37 °C for 60 min and then washed five times with wash buffer. Chromogen A and B were then added and incubated for 10 min at 37 °C, and the inhibition solution was applied. Absorbance was measured at 450 nm, and analysis was performed using EnVision^®^ software (EnVision 2104 Multilabel Plate Reader; PerkinElmer, Waltham, MA, USA) based on a linear curve. Final concentrations of irisin, myonectin, agrin, and myostatin were expressed in ng/mL.

### 2.5. Statistical Analyses

Statistical analysis was performed using Statistica 13.1 software (StatSoft, Inc., Tulsa, OK, USA). Descriptive statistics, including the number of patients, patient percentages, mean, and standard deviation, were used to characterize the study group. The normality of distribution was assessed using the Shapiro–Wilk test. Student’s *t*-test and the Mann–Whitney U-test were used to analyze differences between two groups, while the Kruskal–Wallis test or ANOVA test was used to analyze differences between multiple groups. The correlation analysis was performed using Spearman’s Rho test. The *t*-test for dependent samples and the Wilcoxon test were used to test dependent variables. A statistical significance was attributed to results where the *p*-value was lower than 0.05.

## 3. Results

Detailed information regarding the characteristics of the study group is presented in Table 1.

The results of tests performed on patients before and after rehabilitation are presented in Table 2.

Table 3 shows the relationships between the different groups and myokine levels. A post hoc analysis of statistically significant differences in parameters was performed in the intergroup analysis of many variables (BMI vs. Myostatin). It was shown that there were no statistically significant differences between the analyzed groups.

Table 4 shows the correlations between the levels of selected myokines and the length of hospitalization of COVID-19 patients. A positive correlation with myostatin was demonstrated.

Table 5 shows the correlations between the concentrations of selected myokines and the length of rehabilitation of patients after COVID-19. A positive correlation with myostatin and a negative correlation with myonectin and irisin was demonstrated.

## 4. Discussion

To our knowledge, no studies have yet explored the correlation between selected myokines and the duration of rehabilitation following COVID-19. This study aims to fill this gap and contribute significantly to the advancement of innovative diagnostic and physiotherapeutic strategies for treating and rehabilitating SARS-CoV-2-infected patients. Given the potential link between myokines and the dynamics and consequences of COVID-19, our study focused on analyzing the impact of these proteins on the rehabilitation period for patients recovering from the infection. Existing studies on the endocrine role of skeletal muscle are limited, typically concentrating on the association between myokine levels and the inflammatory response intensity and severity of the COVID-19 course.

The primary goal of rehabilitating patients after COVID-19 is to expedite their return to functional capacity, social life, and work. Evidence underscores the pivotal role of physiotherapeutic interventions in achieving these objectives. Both in-patient and out-patient rehabilitation post-COVID-19 have shown substantial benefits for patients in terms of physical and mental health [34,35,36]. Numerous studies support the effectiveness of rehabilitation programs after COVID-19 [37,38,39,40]. As affirmed by our study, appropriate physiotherapy procedures yield advantages such as enhanced physical performance, pulmonary function, and strength, ultimately contributing to improved quality of life for patients. This study investigated the potential impact of metabolic and muscular changes on the duration of rehabilitation after infection. Additionally, we conducted an analysis to assess whether the length of hospitalization in patients with COVID-19 may affect the characteristics of the cytokine profile in muscle tissue.

Skeletal muscle plays a key role in regulating metabolic processes by secreting cytokines and other peptides. Among the myokines particularly well documented are the anti-inflammatory properties of irisin, myonectin, and the pro-inflammatory effects of myostatin [8,32,33]. The levels of these myokines in patients after COVID-19 may be affected by factors such as increased inflammatory processes causing muscle fiber damage, prolonged bed rest, nutritional disturbances, or the treatment administered. SARS-CoV-2 infection also leads to a highly catabolic state in which significant metabolic changes affect homeostasis and significantly impair myocyte structure, quantity, and function [2,41,42]. The main factor affecting myokine levels is the reduced level of physical activity caused by prolonged inactivity during SARS-CoV-2 infection. Patients hospitalized for COVID-19 are particularly susceptible because they have greater muscle damage [43,44]. Lack of physical activity has a catabolic effect on muscle tissue [45]. Muscle loss may also be a consequence of chronic diseases. One theory suggests that acute sarcopenia may be caused by a stressor such as acute illness, surgery, trauma or burn, or infection, including COVID-19 infection, leading to further muscle loss. According to Aryan et al., physical inactivity during COVID-19 infection was associated with changes in the myostatin pathway [7]. In addition, the patient may experience profound weakness due to mechanical ventilation or the presence of post-intensive care syndrome (PICS). Immobilization has been shown to result in significant changes in muscle cross-sectional area, volume, and mass, promoting metabolic dysfunction and leading to functional impairment [43,44]. To effectively prevent the development of sarcopenia, it is important to know the factors predisposing to it. According to the results of other authors, the risk of sarcopenia associated with an average hospital stay of 11 days due to COVID-19 is 38.4% [46]. Furthermore, persistent inactivity is associated not only with a worse prognosis but also with a higher mortality rate [43]. Numerous studies indicate that the occurrence of sarcopenia is associated with a more severe course of COVID-19, including the need for treatment in an intensive care unit, prolonged hospitalization, and an increased risk of death. There is also a risk of developing an acute form of sarcopenia in patients with COVID-19, especially in the elderly, including due to an increased inflammatory process causing damage to muscle fibers, with the participation of other factors such as prolonged bed rest, eating disorders and increased muscle catabolism [41,42,46].

Myostatin is abundant in skeletal muscle, but also in adipose tissue and heart muscle. Myostatin exerts an inhibitory effect on skeletal muscle by inhibiting cellular metabolic processes. By increasing protein degradation, it promotes muscle atrophy. Myostatin is also a pro-oxidant and causes oxidative stress in skeletal muscle cells. Increased myostatin levels also contribute to the development of sarcopenia, especially in the elderly, in people with cancer, in patients with sepsis, in patients treated in intensive care units, and in patients with chronic obstructive pulmonary disease. High myostatin concentrations also occur in obese patients [1,7,23]. According to the literature, higher myostatin concentration was associated with worse general conditions, lower arterial blood oxygenation, and worse prognosis in patients with chronic obstructive pulmonary disease [47]. In patients with rheumatoid arthritis, high myostatin levels indicated the risk of faster disease progression [48]. Experimental studies indicate a relationship between increased myostatin levels and more severe viral and bacterial infections [49,50]. In the present study, we found a positive correlation between myostatin levels and length of hospital stay in COVID-19. The study also found a significant positive correlation between myostatin levels and the length of rehabilitation, which is the first observation of this kind in the literature. Based on these results, it can be speculated that elevated myostatin levels may affect patient prognosis by increasing the risk of functional impairment and physical limitations after COVID-19 infection.

Irisin reduces the expression of genes related to the intensity of replication of the SARS-CoV-2 virus and increases the expression of genes related to the inhibition of its replication. Epidemiological and biochemical analyses show a relationship between the level of irisin and obesity, noting a decrease in the concentration of this protein when overweight [16,17]. Such a correlation may provide a mechanism explaining the increased susceptibility of people who are overweight to the adverse course of SARS-CoV-2 infection. The relationship between myonectin and the course of COVID-19 has not been clearly established. The protein is known to be involved in the regulation of glucose and lipid metabolism, which is important for patients with metabolic problems caused by COVID-19 [19,20]. In this study, a negative correlation was found between the concentration of irisin and myonectin in patients admitted to the rehabilitation ward after COVID-19 disease and the duration of their rehabilitation. However, taking into account the available literature, no clear link has been identified between the level of irisin and myonectin and the severity of COVID-19, which indicates the need for further research to explain this complex interaction.

### Limitations

The limitation of this study is the lack of repeated measurement of myokine concentrations after rehabilitation, which makes it impossible to assess whether the rehabilitation plan improved the level of selected myokines. The study did not assess factors such as diet and different sleep times of the patients, which could have influenced the course of the study. The limitations of the study may also include the variable starting point of rehabilitation after recovery from COVID-19. However, it was not longer than 12 months, and the analyses performed depending on the different periods of rehabilitation initiation did not show any differences.

## 5. Conclusions

Baseline levels of specific myokines in post-COVID-19 patients could play a crucial role in anticipating the duration of rehabilitation. The duration of hospitalization for the infection may influence myokine levels in patients recovering from COVID-19.

## Figures and Tables

**Table 1:** Characteristics of the study group.

Variable		*n*	%
Sex	Female	91	53.2
	Male	76	46.8
Age	<60 years	46	26.9
	>60 years	121	73.1
Nutritional status (BMI)	18.5–24.99 (norm)	36	21.1
	25.0–29.9 (overweight)	60	35.1
	30.0–34.99 (1st degree obesity).	48	28.1
	35.0–39.99 (2nd degree obesity).	18	10.5
	over 40 (3rd degree obesity)	5	2.9
Hospitalization	Yes	117	68.4
	No	44	25.7
Length of hospitalization	1–5 days	5	2.9
	6–10 days	18	7.6
	11–15 days	31	18.1
	16–20 days	18	10.5
	More than 20 days	50	29.2
Pneumonia during COVID-19 infection	Yes	128	74.8
	No	34	19.9
Oxygen therapy during hospitalization	Yes	106	61.9
	No	38	22.2
The duration of rehabilitation	2–3 weeks	75	43.8
	3–4 weeks	23	13.5
	4–5 weeks	16	9.4
	5–6 weeks	51	29.8
Comorbidities	Diabetes	50	29.2
	Hypertension	113	66.1
	Asthma	19	11.1
	COPD	9	5.3
Smoking status	Yes	15	8.8
	No	146	85.4
Concentration of myokines	Me ± SD		
	Myostatin (ng/mL)	326.8 ± 316.2	
	Agrin (ng/mL)	3.5 ± 3.2	
	Irisin (ng/mL)	13.1 ± 17.9	
	Myonectin (ng/mL)	5.0 ± 4.7	

Legend: *n*—number, BMI—body mass index, COPD—chronic obstructive pulmonary disease; M—mean, SD—standard deviation.

**Table 2:** Relationships between parameters before and after rehabilitation.

Variable	Before Rehabilitation	After Rehabilitation	*p*
	M (±SD)	M (±SD)	
6MWT distance (m)	370.03 (±122.63)	490.47 (±144.26)	<0.001 *
6MWT distance (%predicted).	74.61 (±23.53)	98.44 (±24.89)	<0.001 *
Borg’s scale (6–20)	12.26 (±2.28)	10.39 (±2.61)	<0.001 *
FVC (% predicted)	83.39 (±22.71)	89.71 ± 21.47	0.016 *
FEV1 (% predicted)	86.36 (±24.01)	96.81 (21.46)	<0.001 *
FEV1/FVC (%)	86.32 (13.23)	87.39 (9.91)	0.071
mMRC	2.55 (0.66)	0.73 (0.72)	<0.001 *
FAS scale	28.21 (9.05)	20.68 (6.63)	<0.001 *
Clamping force (kg)	27.23 (10.84)	29.69 (10.91)	<0.001 *

Legend: 6MWT—6 min walk test, M—mean, SD—standard deviation, *p*—statistical significance, * *p* < 0.05.

**Table 3:** Intergroup relationships relative to concentrations of selected myokines.

Variable		Myostatin		Agrin		Irisin		Myonectin	
		M ± SD	*p*	M ± SD	*p*	M ± SD	*p*	M ± SD	*p*
Sex	Female	315.83 ± 301.14	0.878	3.33 ± 3.12	0.362	14.71 ± 18.92	0.08	5.52 ± 5.23	0.166
	Male	339.62 ± 334.60		3.68 ± 3.31		11.07 ± 16.67		4.37 ± 3.96	
Age	<65	318.34 ± 270.99	0.694	4.27 ± 3.75	0.121	14.66 ± 20.28	0.785	5.75 ± 4.95	0.072
	>65	329.83 ± 331.87		3.18 ± 2.91		12.41 ± 17.03		4.72 ± 4.62	
BMI	<25	423.48 ± 400.46	0.042 *	3.25 ± 2.52	0.469	15.31 ± 21.70	0.917	5.23 ± 4.34	0.331
	≥25	374.34 ± 350.33		3.52 ± 2.91		12.61 ± 17.65		4.35 ± 3.90	
	≤30	238.73 ± 199.09		3.57 ± 3.67		12.29 ± 16.27		5.43 ± 5.48	
Hospitalization	Yes	325.96 ± 325.11	0.645	3.23 ± 2.97	0.284	12.12 ± 16.83	0.287	4.92 ± 4.71	0.427
	No	326.21 ± 302.71		4.15 ± 3.79		15.02 ± 19.59		4.94 ± 4.77	
Diabetes	Yes	341.61 ± 299.32	0.548	3.46 ± 3.42	0.530	12.17 ± 17.29	0.581	4.42 ± 4.49	0.142
	No	297.38 ± 326.57		3.51 ± 3.15		13.77 ± 18.58		5.24 ± 4.87	
Hypertension	Yes	309.31 ± 316.51	0.062	3.31 ± 2.97	0.542	13.26 ± 17.77	0.357	5.02 ± 4.93	0.623
	No	374.11 ± 320.39		3.94 ± 3.76		13.29 ± 19.17		4.94 ± 4.38	

Legend: BMI—body mass index, M—mean, SD—standard deviation, *p*—statistical significance, * *p* < 0.05.

**Table 4:** Correlations between the levels of selected myokines and the length of hospitalization of patients in the course of COVID-19.

Variable		R	*p*
Duration of hospitalization	Myostatin	0.24713	0.008 *
	Agrin	0.06777	0.469
	Irisin	0.02055	0.825
	Myonectin	−0.10978	0.241

Legend: R—correlation coefficient, *p*—statistical significance, * *p* < 0.05.

**Table 5:** Correlations between the levels of selected myokines and the duration of rehabilitation of patients after COVID-19.

Variable		R	*p*
Rehabilitation time	Myostatin	0.17618	0.029 *
	Agrin	−0.07789	0.323
	Irisin	−0.24241	0.002 *
	Myonectin	−0.24592	0.001 *

Legend: R—correlation coefficient, *p*—statistical significance, * *p* < 0.05.

## Data Availability

The data that support the findings of this study are available from the corresponding author, (A.M.), upon reasonable request.

## References

[1] Barbalho S.M., Minniti G., Miola V.F.B., Haber J.F.D.S., Bueno P.C.D.S., Haber L.S.d.A., Girio R.S.J., Detregiachi C.R.P., Dall’antonia C.T., Rodrigues V.D. (2023). Organokines in COVID-19: A Systematic Review. Cells.

[2] Filgueira T.O., Castoldi A., Santos L.E.R., de Amorim G.J., de Sousa Fernandes M.S., Anastácio W.d.L.D.N., Campos E.Z., Santos T.M., Souto F.O. (2021). The Relevance of a Physical Active Lifestyle and Physical Fitness on Immune Defense: Mitigating Disease Burden, With Focus on COVID-19 Consequences. Front. Immunol..

[3] Soares M.N., Eggelbusch M., Naddaf E., Gerrits K.H.L., van der Schaaf M., van den Borst B., Wiersinga W.J., van Vugt M., Weijs P.J.M., Murray A.J. (2022). Skeletal muscle alterations in patients with acute COVID-19 and post-acute sequelae of COVID-19. J. Cachexia Sarcopenia Muscle.

[4] Ragab D., Salah Eldin H., Taeimah M., Khattab R., Salem R. (2020). The COVID-19 Cytokine Storm; What We Know So Far. Front. Immunol..

[5] Soares-Schanoski A., Sauerwald N., Goforth C.W., Periasamy S., Weir D.L., Lizewski S., Lizewski R., Ge Y., Kuzmina N.A., Nair V.D. (2022). Asymptomatic SARS-CoV-2 Infection Is Associated with Higher Levels of Serum IL-17C, Matrix Metalloproteinase 10 and Fibroblast Growth Factors Than Mild Symptomatic COVID-19. Front. Immunol..

[6] Di Filippo L., De Lorenzo R., Sciorati C., Capobianco A., Lorè N.I., Giustina A., Manfredi A.A., Rovere-Querini P., Conte C. (2021). Adiponectin to leptin ratio reflects inflammatory burden and survival in COVID-19. Diabetes Metab..

[7] Aryana I., Setiati S., Rini S.S. (2021). Molecular Mechanism of -Acute Sarcopenia in Elderly Patient with COVID-19. Acta Med. Indones..

[8] Pedersen B.K., Åkerström T.C.A., Nielsen A.R., Fischer C.P. (2007). Role of Myokines in Exercise and Metabolism. J. Appl. Physiol..

[9] Pedersen B.K. (2013). Muscle as a secretory organ. Compr. Physiol..

[10] Choi K.M. (2016). The impact of organokines on insulin resistance, inflammation, and atherosclerosis. Endocrinol. Metab..

[11] Coelho-Junior H.J., Picca A., Calvani R., Uchida M.C., Marzetti E. (2019). If my muscle could talk: Myokines as a biomarker of frailty. Exp. Gerontol..

[12] Severinsen M.C.K., Pedersen B.K. (2020). Muscle-Organ Crosstalk: The Emerging Roles of Myokines. Endocr. Rev..

[13] Khan S.U., Ghafoor S. (2019). Myokines: Discovery challenges and therapeutic impediments. J. Pak. Med. Assoc..

[14] Delezie J., Handschin C. (2018). Endocrine Crosstalk Between Skeletal Muscle and the Brain. Front. Neurol..

[15] Boström P., Wu J., Jedrychowski M.P., Korde A., Ye L., Lo J.C., Rasbach K.A., Boström E.A., Choi J.H., Long J.Z. (2012). A *PGC1α*-dependent myokine that drives brown-fat-like development of white fat and thermogenesis. Nature.

[16] Polyzos S.A., Anastasilakis A.D., Efstathiadou Z.A., Makras P., Perakakis N., Kountouras J., Mantzoros C.S. (2018). Irisin in Metabolic Diseases. Endocrine.

[17] de Oliveira M., De Sibio M.T., Mathias L.S., Rodrigues B.M., Sakalem M.E., Nogueira C.R. (2020). Irisin modulates genes associated with severe coronavirus disease (COVID-19) outcome in human subcutaneous adipocytes cell culture. Mol. Cell Endocrinol..

[18] Wu Z., Zhang Z., Wang X., Zhang J., Ren C., Li Y., Gao L., Liang X., Wang P., Ma C. (2021). Palmitoylation of SARS-CoV-2 S protein is essential for viral infectivity. Signal Transduct. Target. Ther..

[19] Seldin M.M., Peterson J.M., Byerly M.S., Wei Z., Wong G.W. (2012). Myonectin (CTRP15), a novel myokine that links skeletal muscle to systemic lipid homeostasis. J. Biol. Chem..

[20] Little H.C., Rodriguez S., Lei X., Tan S.Y., Stewart A.N., Sahagun A., Sarver D.C., Wong G.W. (2019). Myonectin deletion promotes adipose fat storage and reduces liver steatosis. FASEB J..

[21] Schäffler A., Buechler C. (2012). CTRP family: Linking immunity to metabolism. Trends Endocrinol. Metab..

[22] Gros K., Matkovič U., Parato G., Miš K., Luin E., Bernareggi A., Sciancalepore M., Marš T., Lorenzon P., Pirkmajer S. (2022). Neuronal Agrin Promotes Proliferation of Primary Human Myoblasts in an Age-Dependent Manner. Int. J. Mol. Sci..

[23] Consitt L.A., Clark B.C. (2018). The vicious cycle of myostatin signaling in sarcopenic obesity: Myostatin role in skeletal muscle growth, insulin signaling and implications for clinical trials. J. Frailty Aging.

[24] Allen D.L., Hittel D.S., McPherron A.C. (2011). Expression and Function of Myostatin in Obesity, Diabetes, and Exercise Adaptation. Med. Sci. Sports Exerc..

[25] Hittel D.S., Berggren J.R., Shearer J., Boyle K., Houmard J.A. (2009). Increased Secretion and Expression of Myostatin in Skeletal Muscle from Extremely Obese Women. Diabetes.

[26] Order of the President of the National Health Fund No. 172/2021/DSOZ of 18 October 2021. https://baw.nfz.gov.pl/NFZ/tabBrowser/mainPage.

[27] Klok F.A., Boon G.J.A.M., Barco S., Endres M., Geelhoed J.J.M., Knauss S., Rezek S.A., Spruit M.A., Vehreschild J., Siegerink B. (2020). The Post-COVID-19 Functional Status scale: A tool to measure functional status over time after COVID-19. Eur. Respir. J..

[28] Paternostro-Sluga T., Grim-Stieger M., Posch M., Schuhfried O., Vacariu G., Mittermaier C., Bittner C., Fialka-Moser V. (2008). Reliability and validity of the Medical Research Council (MRC) scale and a modified scale for testing muscle strength in patients with radial palsy. J. Rehabil. Med..

[29] Hayata A., Minakata Y., Matsunaga K., Nakanishi M., Yamamoto N. (2016). Differences in physical activity according to mMRC grade in patients with COPD. Int. J. Chron. Obstruct. Pulmon. Dis..

[30] Graham B.L., Steenbruggen I., Miller M.R., Barjaktarevic I.Z., Cooper B.G., Hall G.L., Hallstrand T.S., Kaminsky D.A., McCarthy K., McCormack M.C. (2019). Standardization of Spirometry 2019 Update. An Official American Thoracic Society and European Respiratory Society Technical Statement. Am. J. Respir. Crit. Care Med..

[31] Singh S.J., Puhan M.A., Andrianopoulos V., Hernandes N.A., Mitchell K.E., Hill C.J., Lee A.L., Camillo C.A., Troosters T., Spruit M.A. (2014). An official systematic review of the European Respiratory Society/American Thoracic Society: Measurement properties of field walking tests in chronic respiratory disease. Eur. Respir. J..

[32] Intarakamhang P., Wangjongmeechaikul P. (2013). The assessment of dyspnea during the vigorous intensity exercise by three Dyspnea Rating Scales in inactive medical personnel. Glob. J. Health Sci..

[33] Alhanbali S., AlJasser A., Aboudi O., Alaqrabawi W., Munro K.J. (2023). Establishing the reliability and the validity of the Arabic translated versions of the Effort Assessment Scale and the Fatigue Assessment Scale. Int. J. Audiol..

[34] Udina C., Ars J., Morandi A., Vilaró J., Cáceres C., Inzitari M. (2021). Rehabilitation in adult post-COVID-19 patients in post-acute care with Therapeutic Exercise. J. Frailty Aging.

[35] Wittmer V.L., Paro F.M., Duarte H., Capellini V.K., Barbalho-Moulim M.C. (2021). Early mobilization and physical exercise in patients with COVID-19: A narrative literature review. Complement. Ther. Clin. Pract..

[36] Lugo-Agudelo L.H., Cruz Sarmiento K.M., Spir Brunal M.A., Velásquez Correa J.C., Posada Borrero A.M., Franco L., Ianini R., Lis P., Vélez C., Lugo D. (2021). Adaptations for rehabilitation services during the COVID-19 pandemic proposed by scientific organizations and rehabilitation professionals. J. Rehabil. Med..

[37] Liu K., Zhang W., Yang Y., Zhang J., Li Y., Chen Y. (2020). Respiratory rehabilitation in elderly patients with COVID-19: A randomized controlled study. Complement. Ther. Clin. Pract..

[38] Loboda D., Gibinski M., Wilczek J., Paradowska-Nowakowska E., Ekiert K., Rybicka E., Sarecka-Hujar B., Szoltysek-Boldys I., Zielinska-Danch W., Golba K.S. (2023). Effectiveness of cardiopulmonary rehabilitation after COVID-19 in Poland. Pol. Arch. Intern. Med..

[39] Hermann M., Pekacka-Egli A.M., Witassek F., Baumgaertner R., Schoendorf S., Spielmanns M. (2020). Feasibility and Efficacy of Cardiopulmonary Rehabilitation After COVID-19. Am. J. Phys. Med. Rehabil..

[40] Hockele L.F., Sachet Affonso J.V., Rossi D., Eibel B. (2022). Pulmonary and Functional Rehabilitation Improves Functional Capacity, Pulmonary Function and Respiratory Muscle Strength in Post COVID-19 Patients: Pilot Clinical Trial. Int. J. Environ. Res. Public. Health..

[41] Alizadeh Pahlavani H. (2022). Exercise Therapy for People With Sarcopenic Obesity: Myokines and Adipokines as Effective Actors. Front. Endocrinol.

[42] Tokunbo O., Abayomi T., Adekomi D., Oyeyipo I. (2021). COVID-19: Sitting is the new smoking; the role of exercise in augmenting the immune system among the elderly. Afr. Health Sci..

[43] Michel J.-P., Maggi S., Ecarnot F. (2020). Raising awareness of the needs of older COVID patients after hospital discharge. Aging Clin. Exp. Res..

[44] Stam H.J., Stucki G., Bickenbach J. (2020). European Academy of Rehabilitation Medicine COVID-19 and post intensive care syndrome: A call for action. J. Rehabil. Med..

[45] Kozłowska-Flis M., Rodziewicz-Flis E., Micielska K., Kortas J., Jaworska J., Borkowska A., Sansoni V., Perego S., Lombardi G., Ziemann E. (2021). Short and long-term effects of high-intensity interval training applied alone or with whole-body cryostimulation on glucose homeostasis and myokine levels in overweight to obese subjects. Front. Biosci..

[46] Kirwan R., McCullough D., Butler T., de Heredia F.P., Davies I.G., Stewart C. (2020). Sarcopenia during COVID-19 lockdown restrictions: Long-term health effects of short-term muscle loss. GeroScience.

[47] Wen X., Liu P., Wu H., Zhou X. (2014). Relation between serum myostatin with BMI and PaO_2_/PaCO_2_ in patients with chronic obstructive pulmonary disease. Zhong Nan Da Xue Xue Bao Yi Xue Ban.

[48] Lin J.-Z., Ma J.-D., Yang L.-J., Zou Y.-W., Zhang X.-P., Pan J., Li Q.-H., Li H.-G., Yang Z.-H., Wu T. (2022). Myokine Myostatin Is a Novel Predictor of One-Year Radiographic Progression in Patients with Rheumatoid Arthritis: A Prospective Cohort Study. Front. Immunol..

[49] Grunow J.J., Reiher K., Carbon N.M., Engelhardt L.J., Mai K., Koch S., Schefold J.C., Z’graggen W., Schaller S.J., Fielitz J. (2022). Muscular Myostatin Gene Expression and Plasma Concentrations Are Decreased in Critically Ill Patients. Crit. Care Lond. Engl..

[50] Escobar J., Van Alstine W.G., Baker D.H., Johnson R.W. (2004). Decreased Protein Accretion in Pigs with Viral and Bacterial Pneumonia Is Associated with Increased Myostatin Expression in Muscle. J. Nutr..

